# L-DOPA-therapy in Parkinson’s disease: some personal reflections on L-DOPA therapy from Vienna and Berlin

**DOI:** 10.1007/s00702-023-02692-9

**Published:** 2023-10-05

**Authors:** Peter Riederer, Reinhard Horowski

**Affiliations:** 1 Clinic and Policlinic for Psychiatry, Psychosomatics and Psychotherapy, University Hospital Wuerzburg, Margarete‑Hoeppel‑Platz 1, 97080 Würzburg, Germany; 2 University of Southern Denmark Odense, J.B. Winslows Vey 18, 5000 Odense, Denmark; 3 Antaxios GmbH D14 552, Zum Weiher 44, Michendorf, Germany

**Keywords:** Levodopa, History of L-DOPA-therapy, Dopamine receptor agonists, Monoamine oxidase inhibitors, COMT-inhibitors, Levodopa combination therapies

## Abstract

Dopamine was initially considered as a mere intermediate in the noradrenaline synthesis but was then found to be a neurotransmitter. Its depletion resulted in characteristic symptoms in experimental studies and could be antagonized by DOPA (3,4-dihydroxyphenylalanin), suggesting a similarity to the human disorder Parkinson´s disease (PD) and a therapeutic potential which was successfully exploited from the 1970s on. This was due to the pioneering work of Arvid Carlsson and clinicians around the world who first worked on the breakthrough of L-DOPA therapy and then on its amendment and modification and on alternative therapies for PD patients. All these developments led to the establishment of PD therapy as we know it today. It is characterized by the availability of many different compounds which are mostly employed in combination and by different methods: orally, intravenously, transdermally, subcutaneously, or duodenally. Here, we present without claim of completeness some personal reflections about causal drug developments for PD patients and reflect on some personal interactions with leading clinicians and basic researchers who cooperated with us. Such interactions are crucial for the creation, sometimes serendipitously, of fresh ideas and to further develop existing concepts to make therapeutical progress.

## From alkaloids and other remedies to L-DOPA

Both of us, Peter Riederer and Reinhard Horowski, entered the world of Parkinson’s disease (PD) in the early 1970s. I, Peter Riederer, started my career as a basic researcher in Vienna, Austria, and Reinhard Horowski started his employment at Schering AG in Berlin, Germany. In retrospect this time proved to be the decisive decade for the establishment of modern parkinsonian therapy. In the 1970s, Madopar^®^, which is still the gold standard to treat PD, came to the market, apomorphine, a dopamine receptor agonist, was known to be a potential agent; more dopamine agonists were discovered, monoamine oxidase (MAO)-B-inhibitors had their clinical breakthrough and clinical catechol-O-methyltransferase (COMT)-inhibitors studies were initiated. All these developments paved the way for current mono- and combination therapies to treat PD. The dynamics of the 1960s and 1970s were breathtaking, especially if one considers that traditional PD therapy had not moved much forward for some 150 years.

When James Parkinson’s (1817) published his seminal Essay on the Shaking Palsy in 1817, little was known about the medical condition for which he delivered an excellent clinical picture. It was based on the observation of only six sick persons troubled with movement problems, curbed posture, tremors, shaking and constipation. Parkinson concluded that their suffering was due to a single disease affecting the spinal cord. And while he added a chapter on treatment options in his Essay, little to nothing was known about potential remedies and cures for the afflictive disease he described so accurately. In the nineteenth century, attempts to relieve patients from the syndromes of PD involved treatments to appease motor symptoms. Jean-Martin Charcot treated patients at the Salpetrière Hospital in Paris with hyoscyamine (Ordenstein 1868), an alkaloid isolated from solanaceous plants.

Later a cure based on the extract of belladonna emerged and rose to prominence in the early twentieth century when Bulgarian shepherd and self-taught herbalist Ivan Raev (1876–1938) combined extracts from belladonna roots (extracted with white wine, producing the better-resorbed atropine-tartrate) rather than from the traditionally used belladonna leaves, with *calamus* (dwarf lake iris) roots (against a dry mouth, which is why later on chewing gum was also used) and *charcoal* (to postpone the gastric emptying) to have a more stable and longer-lasting application form (Apostolov and Ivanova 1991). As Queen Elena of Italy, wife of Victor Emanuel III. and mother-in-law of the Tsar of Bulgaria, was severely impressed by the new epidemy of severe cases of postencephalitic Parkinsonism in Italy, she introduced this *Bulgarian cure* in 1934 in Italy and 1937 in Kassel, Germany, where her daughter Mafalda was married to a prince of Hessen. Walter Völler became the first clinical director of the *Königin*-*Elena*-*Klinik* in Kassel, the first hospital in the world fully dedicated to Parkinson´s disease. The treatment developed by Raev, which became known as the *Bulgarian cure*, gradually became the standard therapy for Parkinson´s disease (Foley 2001). Indeed, most drugs for PD were belladonna family plant alkaloids, and (in the 1950ies) empiric use of synthetic anticholinergics and antihistamines that achieved the same effect.

## The emergence of L-DOPA

At the beginning of the twentieth century, another plant, vicia faba, a legume crop with high nutritional value, became a focus of a scientific investigation. It found its way into the laboratories of the Swiss company Hoffmann-La Roche, Basle, at that time an internationally renowned producer of cough syrups and medicines based on medicinal plants.

After the Italian Torquato Torquati had isolated a nitrogen-containing substance from vicia faba (Torquati 1913a, b), Markus Guggenheim (1885–1970), a young chemist at Hoffmann-La Roche, repeated these experiments. By varying extraction methods, he produced crystals and identified the isolated aminoacid as l-dioxyphenylalanine (levodopa; Guggenheim 1913). Interested in its commercial development, Guggenheim tested the new substance in animals without significant results and even ingested a small amount (2.5 g) by himself. He noted that to test its quality I swallowed it, but due to heavy vomiting, the substance is not totality innocuous […] I had to vomit twice, so the substance was not fully adsorbed (Guggenheim 1920; Foley 2001). Tragically, Guggenheim became blind in 1916 after an explosion in his laboratory, an extremely unfortunate event that impaired his creative power. Nevertheless, he continued his work on biogenic amines. Yet despite further investigations of levodopa in animal models and improved manufacturing processes, levodopa was nothing more than a simple chemical without known applications when Guggenheim retired in 1948.

Roughly parallel to the investigations at Hoffmann-La Roche, Barger and Dale (1910) had in 1910 discussed the biological activity of “dopamine” which was at that time only known as 3,4-dihydroxyphenylethylamine, synthesized in the same year by Barger and Ewins (1910) in London. One year later, the Polish chemist Casimir Funk, who subsequently also found the first vitamin and coined the word „vitamin “, synthetized DL-DOPA, the racemate (Funk 1911). In the late 1930s, the German pharmacologist Peter Holtz (1902–1970) worked on the biosynthesis of L-Dopa and discovered that the enzyme dopa-decarboxylase forms dopamine from L-DOPA (Holtz 1939). Subsequent works by Hermann (Hugh) Blaschko (Department of Pharmacology, University Oxford, UK) led to the hypothesis that dopamine could have a physiological role of its own (Bergeret et al. 1957). In the following years, it became evident that dopamine exists in the peripheral nervous system (Blaschko 1957) and ultimately also in the brain of humans – as published by Katharine (Kathleen) Montagu (1957), working at the laboratory of Weil-Malherbe at the Runwell Hospital near London, in 1957, though questioned by Arvid Carlsson. (For a review of these years of dopamine research see for example Hornykiewicz 2002 and Foley 2001).

Nevertheless, it was not clear how all these investigations could be useful for the treatment of PD, a perspective that changed dramatically in 1957, when Swedish pharmacologist Arvid Carlsson and his colleagues from Lund, Sweden,  demonstrated that *3*,*4*-*dihydroxyphenylalanine (*DOPA) reversed the sedative effects of reserpine in rabbits. They also found that the reversal of the reserpine-induced sedation and motor inhibition by DOPA was the consequence of the restauration of depleted dopamine levels in the striatum of his rabbits (Carlsson et al 1957). It was a discovery that opened a new era in brain research and gave hope that central nervous disorders could be treated effectively with new chemical agents.

At first, the discovery of young pharmacologist Arvid Carlsson was met with utter disbelief. Neurophysiologists only knew acetylcholine as a neurotransmitter with its fast electrophysiological effects, with most of them in these days adhering to the *sparks* school, whereas Carlsson’s research contributed to a new *soup* school for our understanding of neurotransmission in the brain.

Arvid Carlsson and his coworkers subsequently developed a fluorescence method for measuring catecholamines in organs (Bertler et al. 1958), based upon earlier developments of Falck and Hillarp for their histochemical studies in animals. Later the so-called Aminco-Bowman spectrofluorometer was designed to quantify fluorescent compounds accurately, and this was a major step forward in the research on monoaminergic neurotransmitters, too. In a 1958 follow-up paper, Carlsson reported that reserpine resulted in an almost complete dopamine depletion in the brain and immobility which was counteracted by the administration of L-DOPA (Carlsson et al. 1958). At the same time, two of his students, Bertler and Rosengren (1959a, b, c), measured high quantities of dopamine in the rabbit striatum, an area almost devoid of noradrenaline and known to be implicated in PD. In a review article in 1959, Carlsson (1959) found the highest dopamine concentrations in dog brains in the striatum (which is part of the basal ganglia that controls motor function).

## First clinical observations

The first investigations of L-DOPA in humans must have been quite disappointing, as no clinical use could be found. This also applies to the German Rudolf Degkwitz jun. (1920–1990), at the Department of Psychiatry, University Frankfurt, Germany. Together with his co-worker Prüll, he documented their oral intake of L-DOPA in July 1958. They started with 2 mg and increased the dose up to 180 mg, but they found no significant effects except phases of heavy sweating and slightly increased blood pressure. Later in 1960, Degkwitz et al. described the interactions of L-DOPA with reserpine, chlorpromazine (a dopamine receptor blocker), iproniazide (a non-specific MAO inhibitor) and Vitamin B6 (Degkwitz et al 1960). They wanted to use L-DOPA against the sedative and extrapyramidal side effects of reserpine and chlorpromazine, which were at that time the only remedies for mental disorders available, in patients with schizophrenia. The effects they observed were indeed no less dramatic than those reported later by Walther Birkmayer and Oleh Hornykiewicz (see Foley 2001). As such, they repeated the experimental studies of Arvid Carlsson and co-workers under clinical conditions. Later, Walther Birkmayer and Oleh Hornykiewicz mentioned that they had dissolved L-DOPA according to the description of Degkwitz et al (1960). Four years after Rudolf Degkwitz, Johannes Hirschmann and Klaus Mayer (1964), two neurologists from the University of Tübingen, repeated their experiments with similar results.

The experiences of Isamu Sano (1924–1975) in Osaka, Japan, were equally interesting. He was familiar with antipsychotic drugs and drug-induced parkinsonism. His group independently studied the distribution of catechol compounds including dopamine in human brains, and in 1959 they reported that dopamine was localized in the extrapyramidal system in high concentrations in the human brain, pointing to extraordinarily high concentrations of dopamine in the putamen, caudate nucleus, pallidum, red nucleus/substantia nigra (Sano et al. 1959; 1960). Sano also treated PD patients with 200 mg of DL-DOPA intravenously and observed the disappearance of marked rigidity and tremor for a short moment of time (some 15–30 min). This led him to conclude that the treatment with DOPA had no practical therapeutic value. He also administered a MAO-inhibitor, JB 516, and in combination with DOPA some patients reported beneficial effects. He published his observations in October 1960, first in Japanese and later in his son’s English translation (Sano 2000; Foley et al. 2000 for review). Isamu Sano observed the effects of DL-DOPA on tremor and rigidity (as tremor at this time was regarded as the main symptom of PD), while Walther Birkmayer later reported beneficial effects mainly on akinesia. In addition, Isamu Sano used DL-DOPA which exerts only 50% of the dose activity compared to L-DOPA.

Equally in 1960, Andre Barbeau (1931–1986), the Director of the Departement de Neurobiologie at the Institut de Recherches Cliniques de Montréal, Canada, discovered in a small study that patients with PD excrete dopamine in the urine in much lower quantities compared with normal subjects (Barbeau 1960, 1961; Barbeau et al. 1961a). He repeated and expanded the study in a total of 30 PD patients and gave them single oral doses of levodopa (100 or 200 mg p.o.) alone or combined with a MAO-I or with α-methyldopa, a decarboxylase inhibitor. Methyldopa given alone or in combination with levodopa increased the tremor, whereas levodopa reduced the rigidity to half. The results were reported in part at the 7th International Congress of Neurology in Rome, Italy, 10–15th September 1961 (Barbeau 1961), and in full a few days later at the Bel-Air Symposium (Barbeau et al.1961b).

## Developments in Vienna and the first clinical trial in Lainz Hospital

The decisive years for the history of L-Dopa therapy began in the late 1950ies in Vienna. At the Pharmacological Institute of the University of Vienna, headed by Franz Brücke, the young pharmacologist Oleh Hornykiewicz (1926–2020) had just returned from a stay in Oxford at the laboratory of Hugh Blaschko and decided to start his own dopamine research in Vienna. After the publication of the occurrence of dopamine in the brain by Montagu, he focused on brain tissue and started his first brain dopamine study using whole rat brain homogenates (Hornykiewicz 2006). He then continued his research on human brains. In late 1960, he and his co-worker Herbert Ehringer published their findings of depletion of dopamine in the caudate nucleus and putamen of patients with PD and postencephalitic parkinsonian (Ehringer and Hornykiewicz 1960). Some 10 km away from the Pharmacological Institute, Walther Birkmayer (1910–1996) was head of the Department of Neurology, Pavillon 11, Lainz-Geriatric Hospital, Vienna, Austria, where he was also taking care of a rather large number of PD patients. He was interested in basic research as well, but because Hornykiewicz had turned down an initial proposal for a research cooperation on serotonin/tryptophan back in March 1958, they were not on good terms. To understand this, one must know that Walther Birkmayer in the 1950s was focusing on the serotonergic system for clinical reasons. He regarded fever, sweating, flushes and several symptoms he witnessed in brain-injured soldiers of World War II as “serotonergic symptoms” (Birkmayer 1951, 1971; Birkmayer and Neumayer 1963; Birkmayer and Pilleri 1965). To elaborate his concept, he treated PD patients in 1957 with serotonin and lysergic acid diethylamide (LSD), a derivative of the ergot alkaloid lysergic acid and preferentially a 5-HT-1-as well as 5-HT-2 serotonin receptor agonist with actions also on dopaminergic D1- and D2-receptors. A dose of up to 50γ was applied dropwise to PD patients. Walter Danielczyk, one of his leading physicians, had to sit aside the patient’s bed even overnight and to watch the patients’ reactions, to document them and to have a look at blood pressure, pulse, respiration, vegetative symptoms, and activity (Peter Riederer, personal communication from Walter Danielczyk, June 2023). Serotonin had negative effects while LSD showed motor activation but could not be used any further due to its hallucinogenic adverse reactions.

When, in November 1960, Oleh Hornykiewicz asked Walther Birkmayer to try L-DOPA in his PD patients, Walther Birkmayer refused to do so due to lack of time (Hornykiewicz 2006). He delayed the proposed trial despite Oleh Hornykiewicz urging him towards doing so a couple of times, but, finally, in spring/summer 1961, 20 patients suffering from post-encephalitic, idiopathic and arteriosclerotic Parkinsonism received single intravenous doses of L-DOPA (50–150 mg) over a period of eight to ten weeks in the Department of Neurology at Pavillon 11 of Lainz-Geriatric Hospital (Birkmayer and Hornykiewicz 1961). The investigations were controlled and monitored by Walther Birkmayer, while L-DOPA was provided for this clinical trial by Oleh Hornykiewicz and later by Alfred Pletscher (Research Department, F. Hoffmann-La Roche & Co, Ltd., Basle, Switzerland). It was the start of an extremely fruitful cooperation, as Alfred Pletscher was an excellent researcher himself with a lot of knowledge on emerging chemical agents and neurotransmitters (Pletscher 1985).

Walther Birkmayer and Oleh Hornykiewicz dissolved L-DOPA according to the description of Degkwitz et al. (1960). Their decision to use L-DOPA in the intravenous form may have been the result of having obtained only a very small amount of this compound; it may also have just been a clever way of handling the problem because in this way they successfully avoided all the problems associated with the administration of an oral application. Because L-DOPA is only poorly water-soluble, they dissolved it in boiling water and injected it when it had cooled down to body temperature as described by Degkwitz et al. (1960). Of note, one of us (Peter Riederer) has repeated this procedure to dissolve L-DOPA and confirmed the molecules integrity (Foley 2001). Much later, and certainly in the early 1970ies, Walther Birkmayer frequently used soluble levodopa, as provided by Hoffmann-La Roche in brown vials, for the treatment of acute akinesia, respectively, Off-phases (Peter Riederer, pers. observation).

The results of the first clinical investigation in Lainz-Geriatric Hospital were breathtaking. Bed-ridden patients, who were unable to sit or to walk, could stand up and started walking and moving around, with some even jumping. Their speech, blurred by palilalia and by unclear articulation became forceful and clear as in a normal person. In short, these patients were able to perform motor activities for a short period of time which could not be produced by any other known drug (Birkmayer and Hornykiewicz 1961). Walther Birkmayer and Oleh Hornykiewicz presented their results at the monthly scientific session of Vienna’s Medical Society on November 10, 1961, and published the results of their study on the same day in the “Wiener Klinische Wochenschrift”. Additionally, a black-and-white movie documenting some representative cases was produced. The movie documented patients who had an impressive reaction to the application of intravenous 50 mg of L-DOPA, including their most famous patient, Lisl. There can be no doubt that this film—which soon became a classic—has helped to convince a lot of colleagues and other clinicians that an impressive therapeutic breakthrough may have been achieved.

## Two decisive amendments

Despite this initial euphoria, the following years from 1962 to 1967 passed without much clinical progress. Although the quantities Hoffmann-La Roche and others could provide at that time were not limitless, many clinicians around the world obtained L-DOPA and experimented with it i.v. and p.o., mostly in very low doses, often in single doses and most often only in a few patients. Their results were not conclusive, and the outcomes of these clinical investigations were mostly much less enthusiastic than the ones reported by Walther Birkmayer and Oleh Hornykiewicz. Obviously, intravenous administration of L-DOPA could not become the application form for long-term therapies. Attempts to administer the drug in an oral form were not very successful either until Walther Birkmayer learned to up-titrate this compound very slowly and on an individual basis, thus avoiding most nausea and all emetic effects. Not surprisingly, the effect of the new therapy was also seen with a lot of skepticism at Hoffmann-La Roche, and Alfred Pletscher was concerned that the documented results at Lainz-Geriatric Hospital could be placebo effects as other groups could not replicate them.

Therefore, Alfred Pletscher proposed to Walther Birkmayer in 1964 to test Ro 4–4602 (benserazide; Burkard et al. 1962), a much stronger decarboxylase inhibitor than α-methyldopa, plus L-Dopa i.v. in PD patients for antihypertensive and antidepressant effects. α-methyldopa was tested as an antihypertensive drug under the assumption that it inhibits the biosynthesis of noradrenaline, and the FDA approved it in December 1962 as an antihypertensive agent. Alfred Pletscher’s hypothesis was that preventing the decarboxylation of levodopa would inhibit the formation of dopamine and thereby inhibit the therapeutic action of levodopa. An ongoing L-Dopa effect would thus suggest that Birkmayer’s results were strongly influenced by placebo effects (Pletscher 1985).

Walther Birkmayer, however, after he had learned that this new molecule blocked or reduced the synthesis of dopamine, decided to test it in *Chorea Huntington* patients who were suffering from an excess of uncontrollable movements, and treated them with the new drug. However, to his great surprise, benserazide made the choreatic movements in these patients not better, but much worse. He thus developed the simple idea that, as a consequence of this observation, a drug that makes Chorea Huntington worse, must make Parkinson’s disease better, and added the new compound to his L-DOPA therapy. Indeed, he observed that his PD patients not only responded clearly better and at a quite lower dosage than before but that at the same time they experienced significantly fewer side effects (Birkmayer and Mentasti 1967; Birkmayer 1969). When Alfred Pletscher listened to Walther Birkmayer´s enthusiastic report on the phone, he is reported to have said initially: “The best placebo effect I have ever seen!”. On second thoughts, however, he went into his laboratory and tested the new compound in rats, only to learn that this decarboxylase inhibitor blocked the L-DOPA metabolism into dopamine only outside the blood–brain barrier, as it was not crossing this barrier. Much more L-DOPA came into the brain where it was decarboxylated into dopamine. As quickly as possible, he sent a paper to the journal *Nature* to have his findings published in late 1967 (Bartholini et al. 1967), i.e., still in the same year as the publication of Walther Birkmayer and Maria Mentasti (though Birkmayer had discovered the value of adding this decarboxylase inhibitor to L-DOPA already two years earlier). Normally, the animal data would have come first as a basis for the clinical study, but here it was the other way round.

In fact, Hoffmann-La Roche undertook a major scientific program to clarify why benserazide increases the L-Dopa effect in Parkinson patients. This resulted in the insight that benserazide hardly penetrates the blood–brain barrier, acting mainly in the periphery—in the intestine, the liver, the heart, the capillaries of the brain, but not in its parenchyma. The most frequent side-effects seen after the intake of high levodopa doses, nausea and vomiting, are attributable to high dopamine concentrations in the periphery (Bartholini and Pletscher 1968). Walther Birkmayer’s observation that the use of benserazide increases the efficacy of levodopa and decreases the severity and the incidence of the side effects, had found its rational explanation.

The second decisive development for the breakthrough of L-DOPA therapy happened in New York. George Cotzias of the Rockefeller Institute of Medical Research and the Brookhaven National Laboratory, New York had studied the clinical picture of chronic manganese poisoning in Chile (Cotzias et al 1964) and had believed that the loss of neuromelanin caused the disease. He administered patients melanin and catecholamine precursors, which eventually led him to DL-DOPA (Lees et al 2015). It is not known whether he knew that in 1901, the German Heinrich Embden from Hamburg has published a paper about “die Krankheit der Braunstein-Müller”, where he had described that exposition to manganese oxide (*Braunstein*) could result in a condition very similar to Parkinson’s disease (Emden 1901). Indeed, George Cotzias saw the cause of Parkinson’s disease in the loss of neuromelanin in the substantia nigra and knew that L-DOPA could condensate into this pigment.

In a following clinical trial, George Cotzias used L-DOPA and slowly increased oral doses to up to 16 g in a series of 28 patients with Parkinsonism (Cotzias and Papavasiliou 1967). Improvement of performance was graded as modest in four, moderate in four, marked in 10 and dramatic in 10. This was usually sustained for periods up to two years. Evidence of toxicity was signaled by a few of the variables monitored. Mental effects included enhanced interest, improved memory, transitory sleeplessness and nervousness. Nausea and vomiting were largely circumvented by slowing the increases in the daily dose. A peripheral dopa-decarboxylase inhibitor (DDI) diminished the therapeutic dose of L-dopa required and eliminated anorexia and nausea in one case. Neurologic side effects consisted of involuntary movements ranging from fleeting to severe (Cotzias et al. 1969).

This therapy offered substantial symptomatic relief, though at the price of frequent adverse effects – mostly of a dopaminergic nature (nausea and emesis), but also, as was to be expected from the non-physiological D-DOPA, blood dyscrasias. (When journalists told Walther Birkmayer that George Cotzias has discovered the L-DOPA therapy, he used to say: “oh no! He just discovered the side effects but I was the person to discover the L-DOPA therapy!”). That George Cotzias had to use DL-DOPA first pointed to a general problem at that time. Pure L-DOPA was very expensive and difficult to produce in higher quantities, with Hoffmann-La Roche having a monopoly for quite a number of years. As a result of the great shortage of L-DOPA, Helmut Vorbrüggen, a chemist from the Schering AG at Berlin, had created their own elegant synthesis which worked very well until a heavy explosion destroyed his laboratory, fortunately at lunch-time when nobody was present (Vorbrüggen and Krolikiewicz 1972). Only much later, microbial methods were developed that made enough L-DOPA available at reasonable prices. Needless to say, until that moment, the difficulty of obtaining L-DOPA became a great incentive to combine this drug with other L-DOPA-sparing molecules, and this became an important pre-occupation of Walther Birkmayer and his co-workers.

In January 1973, Hoffmann-La Roche filed a drug application with 463 Madopar^®^ (L-DOPA/benserazide 4:1) patients, 1,059 patients with levodopa alone, and 154 patients with the 3:2 (ratio L-DOPA/benserazide) combination ratio. It was documented that the combination is as effective as L-Dopa but at 5 times lower daily dosages, that the combination ratio 4:1 is better than the ratio 3:2, that Madopar^®^ is better tolerated in general than L-Dopa, and that gastrointestinal side effects are much reduced with Madopar^®^. Later the same year, Hoffmann La Roche started to market Madopar^®^, which combined L-DOPA and benserazide and rapidly became a great marketing success. Two years later, Sinemet^®^ was approved in the USA, though in this case as a combination of L-DOPA with carbidopa (10:1), another decarboxylase inhibitor developed by Merck, whilst Hoffmann La Roche’s original product was never approved in the USA. Reasons were that high dose benserazide in experiments with rats caused changes in bone-formation. This kind of adverse reaction has, however, never been noted under clinical conditions, not even at high-dose benserazide application.

## Problems with L-DOPA and main challenges to be solved after 1973

L-DOPA monotherapy could be expected to become the gold standard of anti-Parkinsonian treatment because it delivers the missing dopamine to the brain. However, as a drug for oral application, L-DOPA had some disadvantages for regular clinical use: due to its very short half-live, it must be given very frequently (which results in bad compliance), while because of its quite variable bioavailability, it needs an individual titration based upon adverse effects which result from its very narrow therapeutic range; in long term treatment—after a few years of the so-called L-DOPA *honey moon –* motor fluctuations appear. A rather great number of problems with the further development of the L-DOPA therapy such as those established by Walther Birkmayer’s initial study (where this drug was given intravenously) remained to be solved. Whilst L-DOPA as an endogenous compound was the logical product to use due to such disadvantages, without further improvement it would have remained a mere laboratory curiosity. Oleh Hornykiewicz wrote indeed in 1966 in a review paper about dopamine: “The therapeutic value of L-DOPA in human Parkinsonism, however, has not yet been definitively established […] due to unpleasant side effects”.

It is correct that L-DOPA has a number of early adverse events such as nausea, emesis and orthostatic hypotension, to which one must add a very short half-life of about 15 min, a highly variable bioavailability and several metabolic pathways. When used as monotherapy, there were *motor fluctuations* with phases of „*ON*/*OFF* “, i. e. with periods of mobility and immobility following each other. Eventually, the threshold for dyskinesias would also lower, with the consequence of more and more unpredictable *motor complications*. Walther Birkmayer continued to collect new patients and reported with his assistant Maria Mentasti in the Deutsche Ärzteblatt, (1972): “The L-Dopa therapy is cumbersome for a general practitioner … it requires a certain theoretical know-how and true medical decision-making … but also rewarding and challenging for the patient and the doctor. Amongst our 4,000 patients treated by us in the last decade, we had very good results in 40%. A great part of them could resume their work, amongst whom three surgeons. In another 40%, the results obtained were good, but only single symptoms such as posture, speech or walking were clearly improved. In the remaining 20% of patients, treatment with L-DOPA remained without success. The average life-time of the 82 patients not treated with L-DOPA was 9.6 years; 85 patients treated with L-DOPA survived on an average of 18.3 years.” [translated from the German language article, Deutsches Ärzteblatt 1972).

Unfortunately, the short terminal half-life of L-DOPA results in multiple peaks and valleys of its plasma levels over the day; with the progression of PD, the therapeutic window becomes smaller. Early morning akinesia, wearing off and on–off appear as motor fluctuations. Later, with the lowering of the threshold for dyskinesias, motor complications (*peak*-*dose dyskinesias*) appear as well. Therefore, and after the approval of Madopar, the PD community was confronted with a couple of critical therapeutic questions: How can continuous dopaminergic stimulation (CDS) be achieved? How can the effect of L-DOPA be improved further? Which way of administration is the best? Could alternatives to L-DOPA be developed?

## Some amendments of L-DOPA formulations

For pharmacokinetic reasons, minimizing the daily dose of L-DOPA becomes crucial. One method to achieve this is adding a decarboxylase inhibitor, in the ratio of 4 to 1 (benserazide; Madopar^®^), as already established by the Viennese group around Walther Birkmayer. Jerzy Wajsbort, Neurologist at the Kuppat Kholim Lin, one of the major clinical health centers in Haifa, Israel, combined L-DOPA with another decarboxylase inhibitor, carbidopa, in very early, but did not get enough carbidopa to treat enough patients with PD. Therefore, he tried a 10:1 combination and not the 4:1 combination like in Madopar. He was successful with this strategy to treat patients with PD successfully. (Jerzy Wajsbort personal communication with Peter Riederer, 1975).

A first, slow-release form of L-DOPA (Madopar^®^ retard) was not successful because in an unpredictable way, some patients had even a low bioavailability. But a new galenical retard formulation, Madopar^®^ HBS (Hydrodynamically Balanced System), was more promising and developed in the mid-1980s. It was effective in producing a prolonged and stable clinical response and in decreasing nocturnal akinesia. Another equally promising approach was that of duodenal infusions, also pioneered in the 1980s when Kurlan et al. (1986) and Sage et al. (1988) described an intra-duodenal infusion of L-DOPA to reduce motor fluctuations. This approach was further amended, in the early 2000s when an L-DOPA/carbidopa intestinal gel (LCIG, Duodopa^®^) came to the market and, more recently, a triple combination (Levodopa, carbidopa and entacapone, Lecigon^®^, Stada) intestinal gel was developed. All in all, such intestinal gel formulations are very beneficial to patients in the more advanced stages of PD.

## The quest for new compounds and the beginning of mono- and combination therapies in the 1970s and 1980s

Immediately after the approval of Madopar^®^, bromocriptine, derived from ergot, was discovered for PD in 1974 by the group of Donald Calne (Calne et al. 1974). In rat studies, this molecule was a potent inhibitor of nidation, and for this reason, scientists from Sandoz AG in Basle (now Novartis AG) expected it to become a contraceptive; surprisingly it was just the opposite, a pro-fertility drug because of its prolactin-lowering effects. It is a dopaminergic D-2 receptor agonist but a D-1 receptor antagonist. Both, in low and high doses, bromocriptine combined with levodopa was usually more effective than bromocriptine alone.

The first known dopamine agonist, however, is apomorphine which in its structure is close to dopamine (Ernst 1967). It was first synthesized in 1845 by Arppe in Finland. Apomorphine is derived from morphine (which had been isolated from opium by Friedrich Sertürner in 1805) and can be obtained by cooking morphine with a strong acid such as hydrochloric acid or sulfuric acid (Matthiessen and Wright 1869). It was found to no longer possess opiate effects, but to be a strong and potent emetic used very frequently during the 1870s, where it replaced Calomel due to the latter’s known toxicity. The first comprehensive evaluation of the pharmacological effects of apomorphine was undertaken in the doctoral thesis of Siebert (1871) from the University of Dorpat (now Tartu). At lower doses, apomorphine has sedative effects and for this reason has been used in patients with agitation (e. g. from alcohol intoxication) (see also Weil 1884). In 1920, Rudolf Magnus wrote a comprehensive review about apomorphine in which he summarized experiments from Harnack and Feser reporting stereotyped behavior, continuously repeated movements induced by apomorphine in a variety of animal species, a phenomenon nowadays known to indicate a dopamine agonist effect. Subsequently, however, apomorphine was used mostly as an emetic in cases of an intoxication to empty the stomach.

Only in 1951 did Schwab (1951), Amador and Lettvin start to use apomorphine to treat PD patients and two years later, Struppler and von Uexküll (1953) used apomorphine to induce *eine vegetative Umstimmung* (a vegetative re-programming).

It took much longer for apomorphine to be shown to be a strong, but short-acting dopaminergic D-1 and D-2 receptor agonist (see Bevan 1983; Millan et al. 2002; Deleu et al. 2004) with only little affinity for the other dopamine receptor subtypes. As a result of this, it only very rarely causes an impulse control disorder syndrome (ICD) consisting of, e.g., gambling, compulsive shopping, binge eating, hypersexuality, etc. as do other dopamine agonists with a high agonist affinity for D-3 receptors such as pramipexole in the limbic system where they control the reward system.

So, the most beneficial employment of apomorphine was a subcutaneous infusion or via a Penject; it could be live-saving in the latter form in situations of acute akinesia or malignant syndrome in Parkinsonian patients or before or after surgery when oral therapies are not possible.

In 1979 Corsini et al. reported the successful use of subcutaneous apomorphine in combination with domperidone, which blocks dopamine receptors in peripheral neurons and the chemoreceptor trigger zone outside the blood–brain barrier, thereby avoiding peripheral side effects. This was confirmed by a series of experiments by Hardie et al. (1984). Obeso et al. (1986) later summarize the CDS results with s.c. lisuride infusion. Stibe et al. (1987; 1988) reported their results with CDS of lisuride and apomorphine. In 1988, Stibe et al. published their findings that subcutaneous apomorphine was a well-tolerated and beneficial treatment in PD patients with severe on–off fluctuations. More recently, the successful use of continuous subcutaneous infusion of apomorphine in overcoming refractory on–off oscillations in Parkinson’s disease was described (Chaudhuri and Clough 1998).

Another agonist, Lisuride, an 8-alpha-amino-ergoline, was synthetized in 1960 by Zikan and Semonský (1960) from the Institute of Chemistry and Pharmacology in Prague and was first used as a peripheral serotonin-2A/B receptor antagonist for migraine prophylaxis, until Horowski and Wachtel (1976) discovered its strong dopaminergic properties. In contrast to the 8-beta-amino-ergolines such as bromocriptine, pergolide and cabergoline, lisuride is a 5-HT-2A/B receptor antagonist and thus does not cause cardiac valvulopathies, which could be observed with other ergot derivatives (Hofmann et al. 2006). Lisuride—as well as apomorphine—can be used as a single short-lasting injection (with immediate onset of action and terminal half-lives of 15 min, respectively, 50 min). Alternatively, they can be applied via a portable micropump such as the crono-Apo Go pump to achieve continuous dopaminergic stimulation (CDS). The oral lisuride formulation (DoperginR) was approved for the treatment of PD in many European countries from 1983 onwards (Wachtel 1991).

To avoid or at least reduce the side effects of L-DOPA/DDI as well as those of dopaminergic receptor agonists, partial receptor agonists have been developed. These drugs have a lower intrinsic activity than full agonists, allowing them to act either as a functional agonist or a functional antagonist depending on the surrounding levels of naturally occurring neurotransmitter (full agonist) (Lieberman 2004). In PD with a loss of the natural full agonist dopamine, a partial receptor agonist would develop functional agonistic activity.

As such it was hypothesized that extrapyramidal symptoms would be avoided. Schering AG developed terguride (trans-dihydrolisuride), a 5-HT2B-receptor antagonist and dopaminergic receptor partial agonist. This drug had indeed a better side effect profile than full dopamine receptor agonists (Brücke et al.^1987^; Akai et al. 1993; Baronti et al. 1992), but its use in PD had been discontinued because of weak clinical efficacy.

It is worth noting that Arvid Carlsson was also interested in dopamine partial agonists and developed (-)-3PPP (*N-n-propyl-3-(3-hydroxyphenyl) piperidine*, a mixed sigma σ1 and σ2 receptor agonist and D2 receptor *partial agonist*) (Tamminga and Carlsson 2002). In 1992 he wrote to Reinhard Horowski from Schering AG in Berlin and asked for a drug sample of terguride to compare the properties of the two drugs.

Over several decades there has been a discussion about the role of dopamine receptor agonists in the therapy of PD. They could be used as monotherapy early in this condition to postpone the start of L-DOPA-therapy with its long-term complications or to reduce this therapy by combining L-DOPA with a dopamine agonist. However, patients, when given a choice, as a rule prefer L-DOPA over dopamine agonists. Nowadays, piribedil, pramipexol, ropinirole and the rotigotine patch, the first transdermal application invented by Schwarz Pharma in the early 2000s, are preferred dopamine receptor agonists (Horowski and Löschmann 2019).

## Monoamine oxidase (MAO) inhibitors

Nature – or rather, evolution – controls highly active molecules such as dopamine (as well as noradrenaline) not with just one mechanism, but – to be on the safe side – in several ways. In the presynaptic part of a neuron, dopamine is sequestered in vesicles and, once released into the synaptic cleft, it is rapidly bound to the postsynaptic receptor; alternatively, and if there is an excess of these active neurotransmitters, they also bind to presynaptic receptors or will again be taken up into the presynaptic neuron to be recycled (neuronal re-uptake mechanism). Other parts of dopamine can be broken down by two different enzymatic pathways – one by monoamine oxidase (MAO) and the other one by catechol-O-methyl-transferase (COMT). Mary LC Hare (1928) at the Biochemical Laboratory, University of Cambridge, UK, showed that the liver contains “tyramine oxidase” and Hugh Blaschko et al. (1937) suggested that MAO may be important in the catabolism of monoamines in the central nervous system.

Iproniazide, a non-specific inhibitor of MAO, reduces rigidity and tremor to some effect in patients with PD, as shown by Isamu Sano in his early study in Osaka. He also has employed a combination of DL-DOPA with JB-516 (pheniprazine). Walther Birkmayer has shown that tranylcypromine, a MAO inhibitor with amphetamine-like effects, improves rigidity and tremor (Bernheimer et al. 1962). This group also used a number of other MAO-inhibitors with a hydrazine structure and observed severe side effects such as blood pressure crises and liver toxicity due to the hydrazine component of those MAO-inhibitors. Therefore, they did not combine L-DOPA and MAO-inhibitors for many years. In 1968 Johnston reported on substrate and inhibitor selectivity of MAO and proposed the MAO-A and MAO-B subtypes (Johnston 1968).

Another emerging compound of interest was L-deprenyl, initially synthetized by Zoltan Ecseri in Hungary 1962 (patented by Ecseri et al. 1964) and developed by Joseph Knoll and his colleagues as a *psychic energizer* (Knoll et al. 1965). First clinical trials with depressed patients were performed in Budapest using L-deprenyl at doses up to 100 mg/day (Varga and Tringer 1967). From these clinical results, it was known that L-deprenyl has no side effects like liver toxicity or increased blood pressure. In 1973 Birkmayer reported at clinical conferences that patients treated with L-DOPA/benserazide suffered from so-called ON–OFF-phases. Based on analyses of dopaminergic metabolites in urine, Peter Riederer proposed to try a safe MAO-inhibitor. By chance, in November 1973, he visited Merton Sandlers laboratory at Queen Charlottes Maternity Hospital in London to develop a gas chromatographic method to detect 3-methoxy-4-hydroxyphenylglycol (MHPG), the main metabolite of noradrenaline in the brain, in the CSF. Peter Riederer used this opportunity to discuss the above problem with Merton Sandler and on his advice with Moussa BH Youdim, who at this time worked with Graham Smith in Oxford. However, these discussions remained without any conclusive results. In October 1974, Moussa Youdim visited Joseph Knoll, head of the Department of Pharmacology at the Semmelweis University in Budapest to discuss experimental studies with L-deprenyl. On his way back, Moussa Youdim made a stop in Vienna, Austria, as asked by Peter Riederer. Moussa gave a lecture but did not mention PD, On–OFF-phases or the use of L-deprenyl in such clinical situations. In a *symposium of two* in a *Heurigen*-Restaurant (Heuriger = new wine) at Grinzing, Vienna, Peter Riederer asked Moussa Youdim for some L-deprenyl to test it in PD patients with motor fluctuations. Whilst Moussa Youdim initially was not convinced at all by this project, because dopamine would be a MAO-A substrate and L-deprenyl would be a MAO-B-Inhibitor, Peter Riederer convinced him finally with the argument that L-deprenyl is safe (no blood pressure crises, no liver toxicity; dopamine in the human being might be more unspecific as substrate for MAO subtypes) and eventually received 3 g to add it to L-DOPA/benserazide (Foley 2001).

To be on the safe side, Peter Riederer then proposed to Walther Birkmayer to try a dose of 10 mg/day instead of the unspecific dose of 100 mg/day as used by Varga and Tringer (1967). Indeed, this dosage proved to be safe and selective enough to block MAO-B only and improved the action of L-DOPA/benserazide in patients with PD (Birkmayer et al 1975 and 1977). It turned out to be highly effective as a L-DOPA enhancer and was also very popular with the patients, because in addition of being an MAO-B inhibitor, it indeed had stimulant effects as well, being a prodrug to amphetamines. In another approach, Birkmayer and Riederer (1983) published evidence for a beneficial action in a small number of patients treated with a triple combination of L-DOPA/benserazide, lisuride i.v. and L-deprenyl/selegiline (marketed internationally e.g. as Eldepryl^®^, Zelapar^®^ Jumex^®^, etc.), i. e. with the result of a maximum reduction of the amount of L-DOPA to ingest.

They also reported on a prolongation of L-DOPA efficacy in PD in an observational clinical trial (Birmayer et al.^1983^), a finding, which could not be confirmed but is still a matter of (clinical) research and discussion.

Later, John Finberg and Finberg and Youdim (2002) at the Department of Pharmacology TECHNION, Haifa, Israel developed rasagiline, which had no stimulating properties and neither such a metabolite. These MAO-B inhibitors, which in contrast to drugs that acted on MAO-A did not potentiate the effects of tyramine, e.g. from food, had reasonable anti-Parkinson properties when given as monotherapy but could also prolong and increase the effects of L-DOPA when given in combination.

Nowadays, selective, reversible MAO-B-I like safinamide have been developed and are beneficial in the armamentarium to fight PD (Cattaneo et al. 2003; Bette et al.^2018^).

## COMT- inhibitors

In 1957, Julius Axelrod isolated a new enzyme that catalyzes the methylation of adrenaline, noradrenaline and other catecholamines and called it catechol-o-methyltransferase (COMT) (Axelrod et al. 1957). For this enzyme, which inactivates L-DOPA in a similar way as MAO, the same holds true regarding the need for a fast inactivation of the very potent catecholamines and also for L-DOPA, which COMT breaks down into 3-O-methyl-DOPA. This metabolite is very stable and will not easily undergo further metabolism. It has a terminal half-life of about 12 h and very effectively competes with L-DOPA at the neutral amino-acid transporter at the blood–brain barrier, especially when a peripheral decarboxylase inhibitor is administered as well. For this reason, inhibiting its production will greatly improve the anti-Parkinson effects of L-DOPA, as had already been suggested by Arvid Carlsson. First trials with N-butyl-gallate failed due to the toxicity of this drug (Ericsson 1971).

Tolcapone, developed by Hoffmann-La Roche, Basle, Switzerland, inhibits COMT in both the peripheral and central nervous system. Due to the risk of severe side effects, tolcapone is regarded as a second-choice medication nowadays.

A new COMT inhibitor was entacapone (Orion Corporation, Espoo, Finland), usually combined with L-DOPA and benserazide (Stalevo^®^, a combination of L-DOPA (50 mg, 100 mg or 150 mg) with carbidopa (12.5 mg, 25 mg or 37.5 mg) and entacapone (200 mg). When higher doses of L-DOPA still are to be used, as in the case of the intra-duodenal infusion (Duodopa^®^), adding a COMT-inhibitor is really a must because without this, a high-dosed treatment with L-DOPA uses up so many methyl-groups that an increase in serum homocysteine levels occurs causing peripheral neuropathies (Müller and Kuhn 2007). Patients treated with very high dosages of L-DOPA, therefore, definitely always need a COMT inhibitor. More recently, opicapone (Neurocrine Biosciences,Inc; UK.), came to the market (Bial) with the advantage of a single daily administration.

## Amantadine

In 1969 Robert Schwab and his colleagues described the beneficial effects of amantadine in Parkinson’s disease. This was based upon the observation of one of Schwab’s female patients who suffered from Parkinson’s disease, who had taken this drug against her *flu* and who then reported its anti-Parkinson effect. In a subsequent study, in a cohort of 163 patients treated with amantadine (100 mg in a capsule over a period of 5 to 7 days), two thirds of these patients exhibited an improvement of their Parkinsonian symptoms with a good tolerability (Schwab et al. 1969).

Being aware of the studies on amantadine by Schwab et al. (1969), Walter Danielczyk decided to further enlighten the clinical potential of amantadine.HCl (Danielczyk and Korten 1971) and – later—especially amantadine.sulfate. In doing so, Walter Danielczyk developed his  own field of therapy and soon published seminal findings on the use of amantadine.sulfate in the so-called akinetic crises and the akinetic end-stage of PD (Danielczyk 1973). We, Walter Danielczyk and Peter Riederer, did many studies on the combination of L-DOPA/benserazide plus amantadine.sulfate and were surprised to see frequently an increase of the area under the curve of plasma dopa levels, which we could not explain at that time. Danielczyk published one such diagram in one of his publications. Only later did it become evident that amantadine is a good decarboxylase inhibitor (Danysz et al. 2021), thus potentiating the level of L-DOPA in brain. In a review article, Riederer and colleagues (1983) presented biochemical and clinical observations of a triple combination of L-DOPA/benserazide with lisuride i.v. and amantadine, respectively, anticholinergics, another combination of anti-PD medications to further reduce the dose of L-DOPA.

It is worth noting that Danielczyk did not forget that amantadine had originally been developed as an anti-viral agent against influenza (see Danysz et al. 2021). We, therefore, immediately treated ourselves with amantadine.sulfate at any upcoming clinical signs of flu to cope with or ideally avoid the symptoms, and this strategy was very effective. Although scientifically not proven, Müller et al. (2023) have raised the hypothesis that amantadine might have its place to treat the long-covid-syndrome after a SARS-CoV-2 infection. Clinical observations as described above opened the way for amantadine as a therapeutic strategy to treat PD patients and to discover the drug’s mode of action (see for review Kornhuber et al. 1991; Danysz et al 2021).

In the spring of 1986, Johannes Kornhuber visited Peter Riederer in Vienna to discuss the glutamate hypothesis of schizophrenia, which had been put forward in 1980 (Kim et al. 1980). In discussing various topics of mutual interest, we talked about our research at the Dept. Neurology, Lainz-Geriatric Hospital and, of course, we touched the unsolved question about the mode of action of amantadine at “Café Landtmann”. We finally came to the conclusion initiated by Johannes Kornhuber that it might be worth studying the mode of action of amantadine and its relation to the glutamatergic system. Johannes Kornhuber suggested to come to Vienna for a research stay to study this problem, but Peter Riederer had to decline this wish as he was to leave for a CIII-Professorship on Clinical Neurochemistry at the Department of Psychiatry, University Würzburg, Germany in September 1986. If that is so, Johannes Kornhuber said, then I will come to Würzburg! So, we both started research in a completely new environment, and Johannes Kornhuber started his research on the role of glutamate in the pathology of schizophrenia as well as to analyse the MoA of amantadine based on a glutamatergic basis. The publications Kornhuber et al. (1989) and Kornhuber et al. (1991), in which it was shown that memantine and amantadine are N-methyl-D-aspartate receptor antagonists, revolutionized the pharmacology and mechanism of action of these amino-adamantanes, thereby giving a new input for the clinical use of these drugs for Alzheimer disease (memantine) and PD (amantadine).

## Final remarks

Based on the pioneering developments in the 1960s, when intravenous and oral L-DOPA therapy started, new therapeutic concepts evolved in the 1970s and 1980s which reduced L-DOPA side effects and/or prolonged its duration of action. With the development of new, non-invasive, transdermal and duodenal formulations, it is now possible to offer patients individual therapies that improve their quality of life as well.

It is noteworthy that in Vienna, Walther Birkmayer and his clinical staff always tried to treat PD patients on an individual basis by combining a variety of drugs including anti-PD medication and anti-depressive drugs to improve psychic behaviour and psychomotor activity (Birkmayer and Riederer 1983). It was an early focus on a personalized therapy of PD patients, an approach which today is part of precision medicine.

It also is worth noting that very soon after the initial discoveries by neuropharmacologists and their translation to the clinics, the therapeutic progress for the benefit of people suffering from a severe neurodegenerative disease was driven by committed and creative clinicians. The evolution of PD therapy as we know it today is the result of a strong interconnection between clinicians, basic researchers and pharmaceutical companies. It was an extremely fruitful and successful path that should be remembered and embraced again for the benefit of patients.

## References

[CR1] Akai T, Yamaguchi M, Mizuta E, Kuno S (1993). Effects of terguride, a partial D2 agonist, on MPTP-lesioned Parkinsonian cynomolgus monkeys. Ann Neurol.

[CR2] Apostolov M, Ivanova P (1991). Relazioni mediche bulgaro-italiane nel terzo decennio del secolo ventesimo [Bulgary-Italy medical relationships]. Med Secoli.

[CR3] Arppe AE (1845) Ueber eine merkwürdige Veränderung des Morphins durch Schwefelsäure. Liebig’s Annalen der Chemie und Pharmacie 1845;LV:96

[CR4] Axelrod J (1957). O-methylation of epinephrine and other catechols in vitro and in vivo. Science.

[CR5] Barbeau A (1960). Preliminary observations on abnormal catecholamine metabolism in basal ganglia diseases. Neurology.

[CR6] Barbeau A, Murphy CF, Sourkes TL (1961). Excretion of dopamine in diseases of basal ganglia. Science.

[CR7] Barbeau, A. (1961) Biochemistry of Parkinson's disease, In: Proceedings of the 7th International Congress of Neurology 1961, Societa Grafica Romana, Rome, p. 925.

[CR8] Barbeau et al. (1961b) Les catecholamines dans la maladie de Parkinson. In: Monoamines et SNC; Symposium Bel-Air; Geneve 1961. DAJ Editor; 1962:p 247–262

[CR9] Barger G, Dale HH (1910). Chemical structure and sympathomimetic action of amines. J Physiol.

[CR10] Barger G, Ewins AJ (1910). Some phenolic derivatives of β-phenylethylamine. J Chem Soc (London).

[CR11] Baronti F, Mouradian MM, Conant KE, Giuffra M, Brughitta G, Chase TN (1992). Partial dopamine agonist therapy of levodopa-induced dyskinesias. Neurology.

[CR12] Bartholini G, Pletscher A (1968). Cerebral accumulation and metabolism of C14-dopa after selective inhibition of peripheral decarboxylase. J Pharmacol Exp Ther.

[CR13] Bartholini G, Burkard WP, Pletscher A (1967). Increase of cerebral catecholamines caused by 3,4-dihydroxyphenylalanine after inhibition of peripheral decarboxylase. Nature.

[CR14] Bergeret B, Blaschko H, Hawes R (1957). Occurrence of an amine oxidase in horse serum. Nature.

[CR15] Bernheimer H, Birkmayer W, Hornykiewicz O (1962). Verhalten der Monoaminooxydase im Gehirn des Menschen nach Therapie mit Monoaminooxydase-Hemmern. Wien Klin Wschr.

[CR16] Bertler A, Carlsson A, Rosengren E, Waldeck B (1958) A method for the fluorimetric determination of adrenaline, noradrenaline, and dopamine in tissues. Kungl.fysiogr.sällsk.Lund förh., 28: 121–123 10.1111/j.1748-1716.1958.tb01627.x 13617023

[CR17] Bertler A, Rosengren E (1959). Occurrence and distribution of dopamine in brain and other tissues. Experientia.

[CR18] Bertler A, Rosengren E (1959). Occurrence and distribution of catechol amines in brain. Acta Physiol Scand.

[CR19] Bertler A, Rosengren E (1959). On the distribution in brain of monoamines and of enzymes responsible for their formation. Experientia.

[CR20] Bette S, Shpiner DS, Singer C, Moore H (2018). Safinamide in the management of patients with Parkinson's disease not stabilized on levodopa: a review of the current clinical evidence. Ther Clin Risk Manag.

[CR21] Bevan P (1983). Repeated apomorphine treatment causes behavioural supersensitivity and dopamine D2 receptor hyposensitivity. Neurosci Lett.

[CR22] Birkmayer W (1951). Hirnverletzungen.

[CR23] Birkmayer W (1969). Experimentelle Ergebnisse über die Kombinationsbehandlung des Parkinsonsyndroms mit L-Dopa und einem Decarboxylasehemmer (Ro 4–4602). Win Klin Wschr.

[CR24] Birkmayer W (1971). 10 Jahre l-DOPA-Therapie des Parkinsonsyndroms. Wien Klin Wschr.

[CR25] Birkmayer W, Hornykiewicz O (1961). Der L-Dioxyphenylalanin (= DOPA)- Effekt bei der Parkinson-Akinese. Wien Klin Wschr.

[CR26] Birkmayer W, Mentasti M (1967). Weitere experimentelle Untersuchungen über den Catcholaminstoffwechsel bei extrapyramidalen Erkrankungen. Arch Psychiat Nervenkr.

[CR27] Birkmayer W, Pilleri G (1965). Die retikuläre Formation des Hirnstammes und ihre Bedeutung für das vegetativ – affektive Verhalten.

[CR28] Birkmayer W, Riederer P, Youdim MB, Linauer W (1975). The potentiation of the anti-akinetic effect after L-dopa treatment by an inhibitor of MAO-B. Deprenil J Neural Transm.

[CR29] Birkmayer W, Riederer P, Ambrozi L, Youdim MBH (1977). Implications of combined treatment with Madopar and L-Deprenil in Parkinson’s disease. A long-term study. Lancet.

[CR30] Birkmayer W, Riederer P, Calne DB (1983). Effects of Lisuride on motor function psychomotor activity and psychic behavior in parkinson’s disease. Lisuride and other dopamine agonists.

[CR31] Birkmayer W, Knoll J, Riederer P, Youdim MB (1983). (-)-Deprenyl leads to prolongation of L-dopa efficacy in Parkinson’s disease. Mod Probl Pharmacopsychiatry.

[CR32] Birkmayer, W, Mentasti, M (1972) Parkinsonkranke mit L-Dopa behandeln Dtsch Arztebl. 69(12): 686-689

[CR33] Birkmayer W, Neumayer E (1963) Die Wärmeregulation beim postenzephalitischen Parkinsonismus. Nervenarzt. 34: 373 14163215

[CR34] Blaschko H (1957). Metabolism and storage of biogenic amines. Experientia.

[CR35] Blaschko H, Richter D, Schlossmann H (1937). The oxidation of adrenaline and other amines. Biochem J.

[CR37] Brücke T, Danielczyk W, Simányi M, Sofic E, Riederer P (1987). Terguride: partial dopamine agonist in the treatment of Parkinson’s disease. Adv Neurol.

[CR38] Burkard WP, Gey FK, Pletscher A (1962). A new inhibitor of decarboxylase of aromatic amino-acids. Experientia.

[CR39] Calne DB, Teychenne PF, Leigh PN, Bamji AN, Greenacre JK (1974). Treatment of parkinsonism with bromocriptine. Lancet (London, England).

[CR40] Carlsson A (1959). The occurrence, distribution and physiological role of catecholamines in the nervous system. Pharmacol Rev.

[CR41] Carlsson A, Lindqvist M, Magnusson T (1957). 3,4-Dihydroxyphenylalanine and 5-hydroxytryptophan as reserpine antagonists. Nature.

[CR42] Carlsson A, Lindqvist M, Magnusson T, Waldeck B (1958). On the presence of 3-hydroxytyramine in brain. Science.

[CR43] Cattaneo C, Caccia C, Marzo A, Maj R, Fariello RG (2003). Pressor response to intravenous tyramine in healthy subjects after safinamide, a novel neuroprotectant with selective, reversible monoamine oxidase B inhibition. Clin Neuropharmacol.

[CR44] Chaudhuri KR, Clough C (1998). Subcutaneous apomorphine in Parkinson’s disease. BMJ.

[CR45] Corsini GU, Del Zompo M, Gessa GL, Mangoni A (1979). Therapeutic efficacy of apomorphine combined with an extracerebral inhibitor of dopamine receptors in Parkinson’s disease. Lancet.

[CR46] Cotzias G, Papavasiliou P, Miller S (1964). Manganese in Melanin. Nature.

[CR47] Cotzias GC, Papavasiliou PS, Gellene R (1969). Modification of Parkinsonism–chronic treatment with L-dopa. N Engl J Med.

[CR48] Cotzias G, Papavasiliou P (1967) Therapeutic studies of parkinsonian patients: long-term studies of d,l- and l-DOPA. Read before the Second International Congress of Neuro-Ophtalmalogy. 1967-9-19. Montreal

[CR49] Danielczyk W (1973). Die Behandlung von akinetischen Krisen. Med Welt.

[CR50] Danielczyk W, Korten JJ (1971). Die Wirkung von Amantadin. HCl allein undin Kombination mit L-DOPA bei Morbus Parkinson. Wr Med Wschr.

[CR51] Danysz W, Dekundy A, Scheschonka A, Riederer P (2021). Amantadine: reappraisal of the timeless diamond-target updates and novel therapeutic potentials. J Neural Transm (vienna).

[CR52] Degkwitz R, Frowein R, Kulenkampff C, Mohs U (1960) [On the effects of L-dopa in man and their modification by reserpine, chlorpromazine, iproniazid and vitamin B6]. Klin Wochenschr. 1;38:120–3. German. doi: 10.1007/BF02189076. PMID: 13815400 10.1007/BF02189076 13815400

[CR53] Deleu D, Hanssens Y, Northway MG (2004). Subcutaneous apomorphine : an evidence-based review of its use in Parkinson’s disease. Drugs Aging.

[CR54] Ecseri Z, Müller M, Knoll J, Somfai E (1964) Hungarian Patent Nr. 151090

[CR55] Ehringer H, Hornykiewicz O (1960). Verteilung von Noradrenalin und Dopamin (3-Hydroxytyramin) im Gehirn des Menschen und ihr Verhalten bei Erkrankungen des extrapyramidalen systems. Klin Wschr.

[CR56] Emden H (1901) Zur Kenntnis der metallischen Nervengifte. Über die chronische Manganvergif tung der Braunsteinmüller. Dtsch med Wschr. 795.

[CR57] Ericsson AD (1971). Potentiation of the L-dopa effect in man by the use of catechol-*O*-methyltransferase inhibitors. J Neurol Sci.

[CR58] Ernst AM (1967). Mode of action of apomorphine and dexamphetamine on gnawing compulsion in rats. Psychopharmacologia.

[CR59] Finberg JP, Youdim MB (2002). Pharmacological properties of the anti-Parkinson drug rasagiline; modification of endogenous brain amines, reserpine reversal, serotonergic and dopaminergic behaviours. Neuropharmacology.

[CR60] Foley P, Mizuno Y, Nagatsu T, Sano A, Youdim MBH, McGeer P, McGeer E, Riederer P (2000). The L-Dopa story — an early lapanese contribution. Parkinsonism Relat Disord.

[CR61] Foley PB (2001) *Beans*, *Roots* and Leaves: a history of the chemical therapy of parkinsonism. tectum 15641199

[CR62] Funk C (1911). Synthesis of dl-3:4-dihydroxyphenylalanine. J Chem Soc.

[CR63] Guggenheim M (1913). Dioxyphenylalanin, eine neue Aminosäure aus Vicia faba. Hoppe-Seylers Zeitschrift Für Physioslogische Chemie.

[CR64] Guggenheim M, Czapek NF, Parnas J (1920). Die biogenen Amine und ihre Bedeutung für die Physiologie und Pathologie des pflanzlichen und tierischen Stoffwechsels. Monographien aus dem Gesamtgebiet der Physiologie der Pflanzen und der Tiere.

[CR65] Hardie RJ, Lees AJ, Stern GM (1984). On-off fluctuations in Parkinson’s disease. A clinical and neuropharmacological study. Brain.

[CR66] Hare ML (1928). Tyramine oxidase: a new enzyme system in liver. Biochem J.

[CR67] Hirschmann J, Mayer K (1964). Neue Wege zur Beeinflussung extrapyramidalmotorischer Störungen. Arzneimittel-Forsch.

[CR68] Hofmann C, Penner U, Dorow R, Pertz HH, Jähnichen S, Horowski R, Latté KP, Palla D, Schurad B (2006). Lisuride, a dopamine receptor agonist with 5-HT2B receptor antagonist properties: absence of cardiac valvulopathy adverse drug reaction reports supports the concept of a crucial role for 5-HT2B receptor agonism in cardiac valvular fibrosis. Clin Neuropharmacol Mar-Apr.

[CR69] Holtz P (1939). Dopadecarboxylase. Naturwissenschaften.

[CR70] Hornykiewicz O (1966). Dopamine (3-hydroxytyramine) and brain function. Pharm Rev.

[CR71] Hornykiewicz O (2002). L-DOPA: from a biologically inactive amino acid to a successful therapeutic agent. Amino Acids.

[CR72] Hornykiewicz O (2006). The discovery of dopamine deficiency in the parkinsonian brain. J Neural Transm Suppl.

[CR74] Horowski R, Löschmann PA (2019). Classical dopamine agonists. J Neural Transm (vienna).

[CR75] Horowski R, Wachtel H (1976). Direct dopaminergic action of lisuride hydrogen maleate, an ergot derivative, in mice. Eur J Pharmacol.

[CR76] Johnston JP (1968). Some observations upon a new inhibitor of monoamine oxidase in brain tissue. Biochem Pharmacol.

[CR77] Kim JS, Kornhuber HH, Schmid-Burgk W, Holzmüller B (1980). Low cerebrospinal fluid glutamate in schizophrenic patients and a new hypothesis on schizophrenia. Neurosci Lett.

[CR78] Knoll J, Ecseri Z, Kelemen K, Nievel J, Knoll B (1965). Phenylisopropvlmethyl propinylamine (E-250) a new psychic energizer. Arch Int Pharmacodyn Titér.

[CR79] Kornhuber J, Bormann J, Retz W, Hübers M, Riederer P (1989). Memantine displaces [3H]MK-801 at therapeutic concentrations in postmortem human frontal cortex. Eur J Pharmacol.

[CR80] Kornhuber J, Bormann J, Hübers M, Rusche K, Riederer P (1991). Effects of the 1-amino-adamantanes at the MK-801-binding site of the NMDA-receptor-gated ion channel: a human postmortem brain study. Eur J Pharmacol.

[CR81] Kurlan R, Rubin AJ, Miller C, Rivera-Calimlim L, Clarke A, Shoulson I (1986). Duodenal delivery of levodopa for on-off fluctuations in parkinsonism: preliminary observations. Ann Neurol.

[CR82] Lees AJ, Tolosa E, Olanow CW (2015). Four pioneers of L-dopa treatment: Arvid Carlsson, Oleh Hornykiewicz, George Cotzias, and Melvin Yahr. Mov Disord.

[CR83] Lieberman JA (2004). Dopamine partial agonists. CNS Drugs.

[CR84] Magnus R (1920). Apomorphin, Apocodein, Ipecacuanha-Alkaloide, Handbuch der Experimentellen Pharmakologie.

[CR85] Matthiessen A, Wright CRA. (1869). Researches into the Chemical Constitution of the Opium Bases. Part I. On the Action of Hydrochloric Acid on Morphia. Proceedings of the Royal Society of London Vol 17 (1868–1869), 455–460.

[CR86] Millan MJ, Maiofiss L, Cussac D, Audinot V, Boutin JA, NewmanTancredi A (2002). Differential actions of antiparkinson agents at multiple classes of monoaminergic receptor. I. A multivariate analysis of the binding profiles of 14 drugs at 21 native and cloned human receptor subtypes. J Pharmacol Exp Ther.

[CR87] Montagu KA (1957). Catechol compounds in rat tissues and in brains of different animals. Nature.

[CR88] Müller T, Kuhn W (2007). Neurotoxicity of levodopa: treatment-associated homocysteine increase. Nat Rev Neurol.

[CR89] Müller T, Riederer P, Kuhn W (2023). Aminoadamantanes: from treatment of Parkinson’s and Alzheimer’s disease to symptom amelioration of long COVID-19 syndrome?. Expert Rev Clin Pharmacol.

[CR90] Obeso JA, Luquin MR, Martínez-Lage JM (1986). Lisuride infusion pump: a device for the treatment of motor fluctuations in Parkinson’s disease. Lancet.

[CR91] Ordenstein L (1868) Paralysie Agitante. A. Delahaye, Libraire-Editeur, Paris

[CR92] Parkinson J (1817) An Essay on the Shaking Palsy. Printed by Whittingham and Rowland for Sherwood, Neely and Jones, London

[CR93] Pletscher A, Riederer P, Umek H (1985). Die Geburt von Madopar: Ratio und Fortuna. L-Dopa substitution der Parkinson-Krankheit.

[CR94] Riederer P, Reynolds GP, Danielczyk W, Jellinger K and Seemann D (1983) Desensitization of Striatal Spiperone-Binding Sites by Dopaminergic Agonists in Parkinso’s Disease. In: Lisuride and Other Dopamine Agonists. DB Calne et al. (Editor), Raven Press. New York, pp 375 - 381

[CR95] Sage JI, Trooskin S, Sonsalla PK, Heikkila R, Duvoisin RC (1988). Long-term duodenal infusion of levodopa for motor fluctuations in parkinsonism. Ann Neurol.

[CR96] Sano I, Gamo T, Kakimoto Y, Taniguchi K, Takesada M, Nishinuma K (1959). Distribution of catechol compounds in human brain. Biochim Biophys Acta.

[CR97] Sano I, Taniguchi K, Gamo T, Takesada M, Kakimoto Y (1960). Die Katechinamine im Zentralnervensystem. Klin Wochenschr..

[CR98] Sano A (2000) Biochemistry of the extrapyramidal system. Parkinsonism Relat Disord 6:3–6. (original: Sano I. (1960) Shinkei Kennkyu No Shinpo, Advances in Neurological Sciences. (ISSN 0001–8724)5:42–48. Japanese) 10.1016/s1353-8020(99)00046-2 18591145

[CR99] Schwab RS, Amador LV, Lettvin JY (1951). Apomorphine in Parkinson’s disease. Trans Am Neurol Assoc.

[CR100] Schwab RS, England AC, Poskanzer DC, Young RR (1969). Amantadine in the treatment of Parkinson’s disease. JAMA.

[CR101] Sertürner F (1805). Darstellung der reinen Mohnsäure (Opiumsäure) nebst einer chemischen Untersuchung des Opiums mit vor- züglicher Hinsicht auf einen darin neu entdeckten Stoff und die dahin gehörigen Bemerkungen. Journal der Pharmacie *14*, 1. Stück.

[CR102] Siebert V (1871) Untersuchungen über die physiologischen Wirkungen des Apomorphin. Inaugural-Dissertation zur Erlangung der Doctorgrades. Dorpat, Druck von Heinrich Laakmann, 1871

[CR103] Stibe C, Lees A, Stern G (1987). Subcutaneous infusion of apomorphine and lisuride in the treatment of parkinsonian on-off fluctuations. Lancet.

[CR104] Stibe CM, Lees AJ, Kempster PA, Stern GM (1988). Subcutaneous apomorphine in parkinsonian on-off oscillations. Lancet (London, England).

[CR105] Struppler A, Von Uexkull T (1953). Studies of mechanism of action of apormorphine on Parkinson’s tremor. Z Klin Med.

[CR106] Tamminga CA, Carlsson A (2002). Partial dopamine agonists and dopaminergic stabilizers, in the treatment of psychosis. Curr Drug Targets CNS Neurol Disord.

[CR107] Torquati T (1913). Über die Gegenwart einer stickstoffhaltigen Substanz in den Keimlingen von Vicia faba. Arch Di Pharmacol Sperimentale.

[CR108] Torquati T (1913). Über die Gegenwart einer stickstoffhaltigen Substanz in den grünen Hülsen von Vicia faba. Arch Di Pharmacol Sperimentale.

[CR109] Varga E, Tringer L (1967). Clinical trial of a new type promptly acting psychoenergetic agent (phenylisopropylmethylpropinyl-HCl. E-250). Acta Med Acad Sci Hung.

[CR110] Vorbrüggen H, Krolikiewicz K (1972). Eine neue Synthese des 3-(3.4-Dihydroxy-phenyl)-L-alanins (L-DOPA [A new synthesis of 3-(3.4-dihydroxyphenyl)-L-alanine (L-DOPA)]. Chem Ber.

[CR111] Wachtel H (1991). Antiparkinsonian dopamine agonists: a review of the pharmacokinetics and neuropharmacology in animals and humans. J Neural Transm Park Dis Dement Sect.

[CR112] Weil E (1884). De l´apomorphine dans certain troubles nerveux. Lyon Medical.

[CR113] Zikán V, Semonský M (1960). Mutterkornalkaloide XVI. Collections.

